# Construction of an artificial symbiotic community using a *Chlorella*–symbiont association as a model

**DOI:** 10.1111/j.1574-6941.2007.00434.x

**Published:** 2008-02

**Authors:** Masato Imase, Keiji Watanabe, Hideki Aoyagi, Hideo Tanaka

**Affiliations:** 1 Graduate School of Life and Environmental Science, University of Tsukuba Tsukuba, Ibaraki, Japan; 2 Ibaraki Kasumigaura Environmental Science Center, Tsuchiura Ibaraki, Japan; 3 Tsukuba Industrial Liaison and Cooperative Research Center, University of Tsukuba Ibaraki, Japan

**Keywords:** *Chlorella*, adhesion, symbiosis, sheath, divalent cation, polysaccharide

## Abstract

*Chlorella sorokiniana* IAM C-212 produces a polysaccharide gel, termed a sheath, under photoautotrophic conditions. The *C. sorokiniana* sheath is a suitable habitat for several symbiotic microorganisms because it ensures close proximity between the *C. sorokiniana* and symbionts. In this study, we established a method for increasing the volume of the sheath produced by *C. sorokiniana*, and proposed a method for constructing artificial communities of *Chlorella* and symbiotic microorganisms. The *C. sorokiniana* sheath was increased by addition of calcium chloride solution. The sheath resulted in coflocculation of *C. sorokiniana* and the associated symbiotic bacteria, thus strengthening the bacterial–*Chlorella* symbiotic association. An application of this technique was demonstrated by constructing a complex of *C. sorokiniana* and a propionate-degrading bacterium (PDS1). Although propionate inhibited the growth of axenic *C. sorokiniana*, the *C. sorokiniana*–PDS1 complex showed good growth in a medium containing a high concentration of propionate.

## Introduction

Symbiotic associations of microorganisms have various applications and are being used efficiently in many fields such as fermented food industries and wastewater treatment. However, their utilization has been based solely on empirical knowledge owing to the complexity of the symbiotic interactions. In other words, application of natural symbiotic associations has been limited.

Microorganisms in natural ecosystems coexist with, and live in symbiotic relationships with, other organisms. Examples of such symbiotic associations include plant root–microorganism (Hirsch *et al.*, 2001; Whipps, 2001), termite–enterobacterium (Brune & Friedrich, 2000; Ohkuma, 2003) and lichens (Ahmadjian & Jacobs, 1981). These natural symbiotic relationships are usually very stable for long periods of time. In natural environments, close proximity is very important for maintaining the symbiotic associations. Thus, some microorganisms have acquired the capacity for chemotaxis, mobility (Paerl, 1982; Paerl & Galluci, 1985) and adhesion with the host (Koblier *et al.*, 1981; Skvortsov & Ignatov, 1998).

Cyanobacteria produce polysaccharide gels that form a sheath. The sheath helps cyanobacteria to attach to other organisms (Robins *et al.*, 1986; Gantar *et al.*, 1995), and vice versa (Caldwell, 1977; Caldwell & Caldwell, 1978). In cyanobacteria–bacteria associations, the cyanobacteria provide photosynthetic products to the heterotrophic bacteria. By contrast, the heterotrophic bacteria use the oxygen produced by the cyanobacteria to degrade organic compounds, thereby maintaining the nitrogenase activity of the cyanobacteria (Paerl & Galluci, 1985; Paerl, 1988). This mutual relationship is facilitated by the environment created by the cyanobacterial sheath. Microalgae also produce a sheath. It was reported that this sheath was related to formation of algal cell aggregations in aquatic ecosystems (Leppard, 1995). Uptake of the metal by microalgal cells (Knauer *et al.*, 1997; Lombardi *et al.*, 2002) and the symbiotic association with bacteria (Croft *et al.*, 2005) have been reported. However, there have been few studies on the relationship between the microalgal sheath and symbiosis. *Chlorella* is a genus of unicellular green algae belonging to the phylum *Chlorophyta*, and members of the genus generally inhabit the hydrosphere. There are several reports of symbiotic associations involving this genus, such as *Chlorella*–*Paramecium* (Summerer *et al.*, 2007) and *Chlorella*–hydra (Rahat & Reich, 1986). However, there are few reports on symbionts of *Chlorella*. The sheath produced by *Chlorella* and their chelating properties have been discussed (Kaplan *et al.*, 1987). However, the relationship between the *Chlorella* sheath and symbionts has not been studied. In our earlier studies, we analysed symbiotic associations in *C. sorokiniana* cultures. The results showed that *C. sorokiniana* and its symbionts adhered together, while some binding through the sheath produced by *C. sorokiniana* was also observed (Watanabe *et al.*, 2005). The results also showed that one of the symbionts (CSSB-3) in the algal sheath promotes growth of *C. sorokiniana*, but the growth-promoting factor was not fully resolved.

Some previous reports have suggested the importance of the sheath in symbiotic systems. In this study, a method for constructing a close and stable artificial complex of microorganisms using an algal sheath was developed.

In our previous work, the mechanism of sheath formation was studied by analysing the composition of the sheath produced by *C. sorokiniana* IAM C-212. The results showed that the major components of the sheath were carbohydrate, protein and metal cations that were found in the cultivation media (Watanabe *et al.*, 2006). It was reported previously that metal cations affect the characteristics and productivity of extracellular polysaccharides (Bonet *et al.*, 1993; Parker *et al.*, 1996). In another study, it was shown that algal polysaccharides absorb metal ions (Kaplan *et al.*, 1988; Mehta & Gaur, 2001). Based on these reports, we presented a hypothesis that the sheath of *C. sorokiniana* was composed of a metal and algal photosynthate.

Although axenic *C. sorokiniana* is very efficient in wastewater treatment, it cannot degrade propionate contained in high-strength organic wastewater. To complement the ability of *C. sorokiniana*, a mixed culture system of *C. sorokiniana* and *Rhodobacter sphaeroides* was studied (Ogbonna *et al.*, 2000). However, *R. sphaeroides* requires extraneous nutrients for its growth. Furthermore, immobilization of the microorganisms is important for their practical application in wastewater treatment, because it allows the biomass to settle for harvesting of sludge or for retaining high-density biomass for further processing (Mallick, 2002). In the present study, we establish a simple method of constructing a *Chlorella*–bacteria complex that can degrade propionate without addition of extraneous nutrients.

## Materials and methods

### Microorganisms

An axenic strain of *C. sorokiniana* IAM C-212 made axenic by the addition of antibiotics (Watanabe *et al.*, 2005) and one of its bacterial symbionts (CSSB-3) isolated from the contaminated strain were used. Strain CSSB-3 (AB167383) showed 98.6% 16S rRNA gene sequence similarity to *Microbacterium trichotecenolyticum* (Y17240), belonging to the high-G+C Gram-positive bacteria (Watanabe *et al.*, 2005).

### Cultivation of *Chlorella*

Preculture of *C. sorokiniana* was carried out according to the method described by Ogbonna *et al.* (1995). Subculturing was carried out every 4 weeks by transferring 5 mL of the precultured broth into a 500-mL Erlenmeyer flask containing 200 mL MM4N medium composed of the following salts (per litre of distilled water): 5.0 g KNO_3_, 1.25 g KH_2_PO_4_, 0.1 g K_2_HPO_4_, 2.5 g MgSO_4_·7H_2_O, 1.8 g of NaCl, 0.04 g FeSO_4_·7H_2_O, 0.03 g EDTA and 1 mL A_5_ solution (containing, per litre of distilled water, 2.86 g H_3_BO_4_, 1.81 g MnCl_2_·4H_2_O, 0.22 g ZnSO_4_·7H_2_O, 0.08 g CuSO_4_·5H_2_O and 0.021 g Na_2_MoO_4_·2H_2_O), and incubating on a rotary shaker (120 r.p.m.) at 30 °C under continuous illumination (50 μmol m^−2^ s^−1^). The preculture was done by inoculating Roux flasks containing 1 L MM4N medium with 10 mL of the stock culture. It was then incubated at 37 °C for 2 weeks under continuous illumination (360 μmol m^−2^ s^−1^). Aeration and mixing were achieved by sparging air enriched with 5% CO_2_ through a glass-ball filter (Kinoshita Rika, Tokyo, Japan), which was inserted to the bottom of the Roux flask, at 0.3 v v^−1^ min^−1^ (vvm).

### Preparation of *Chlorella* culture broth for saccharide aggregation tests

*Chlorella* culture broth was prepared by cultivating *C. sorokiniana* under photoautotrophic conditions for 2 weeks. The culture broth was centrifuged (5000 ***g***, for 15 min at 4 °C), and the supernatant was filtered through a sterile 0.2-μm pore size filter (Millex GV, Millipore, Billerica, MA). Five grams of Chelex-100 cation exchange resin (Nippon Bio-Rad Laboratories, Tokyo, Japan) was added to 100 mL of this filtrate. It was gently shaken for 1 h to remove cations from the filtrate. Residual metals in Chelex-treated filtrate were assayed using a sequential plasma spectrometer (ICPS-8100, Shimazu, Shiga, Japan). They comprised the following (mg L^−1^): Na, <0.1; K, <0.1; Mg, 0.1; Ca, <0.05; Mn, <0.05; Fe, 0.2; Cu, <0.1; Zn, 0; Mo, <0.05.

### Saccharide aggregation with metallic ions

The metals used in aggregation tests were aqueous solutions of KCl, NaCl, MgCl_2_·6H_2_O, CaCl_2_·2H_2_O and SrCl_2_·6H_2_O. Each chloride salt was dissolved in MilliQ water at various concentrations. Five hundred microlitres of the filtered *Chlorella* culture broth and required volumes of the metal solutions were mixed to yield the desired final concentrations (0, 1, 2, 5, 10, 20, 50 mM). After incubation at 25 °C for 15 min, the mixtures were centrifuged at 12 000 ***g*** for 10 min at 4 °C. The pellet was washed five times in 1 mL of each concentration of the metals and then suspended in 500 μL of 50 mM EDTA. The total amount of carbohydrate in the pellet was determined using the phenol/sulphuric acid method with glucose as a standard (Dubois *et al.*, 1956). Results are expressed in mg equivalent of glucose per litre of *Chlorella* culture broth. These experiments were performed in triplicate and the results are expressed as the mean±SE.

### Flocculation and cultivation of axenic *Chlorella*

Flocculated *C. sorokiniana* culture was examined after addition of 50 mM CaCl_2_ to the medium. The precultured *C. sorokiniana* (10 mL) was inoculated to a Roux flask containing 490 mL MM4N medium and incubated at 37 °C for 10 days under continuous illumination. The light intensity, aeration and mixing were achieved as described above. After 3 days of cultivation, 10 mL of 2.5 M CaCl_2_ aqueous solution was added to the culture broth through a 0.2-μm pore size filter. A culture to which 10 mL of distilled water was added was used as the control. Growth of *C. sorokiniana* was monitored by cell counting under a microscope, after addition of 50 mM EDTA to disintegrate cell flocculation. The amount of dissolved polysaccharide in *C. sorokiniana* culture broth was determined as follows. Algal cells were removed by centrifugation and 3 mL of the supernatant was obtained. Seven millilitres of cold 100% ethanol was added to the supernatant, and this was then incubated overnight at 4 °C. After incubation, the mixture was centrifuged at 8000 ***g*** for 10 min to precipitate the polysaccharides. The polysaccharide pellet was washed twice with 80% (v/v) ethanol and finally with 100% ethanol. After evaporating the residual alcohol under vacuum, the polysaccharide pellet was dissolved in an appropriate volume of distilled water to adjust the sugar concentration. Determination of the total saccharide content was examined using the phenol/sulphuric acid method with glucose as a standard.

### Coflocculation of *Chlorella* and the symbiont

The symbiont was precultured in diluted Luria-Bertani (DLB) medium (per litre distilled water: 1.0 g peptone, 0.5 g yeast extract and 1.0 g NaCl, pH 6.8) at 30 °C for 24 h and used after washing in sterile saline solution (0.85% NaCl).

*Chlorella sorokiniana* was cultured axenically in a 500-mL Roux flask as described above and incubated at 37 °C. After 3 days of cultivation, 10 mL of washed cultures (1.0 × 10^6^ cells mL^−1^) of symbiotic bacteria (CSSB-3) was inoculated to *C. sorokiniana* culture followed by addition of 10 mL of 2.5 M CaCl_2_ solution to yield a final concentration of 50 mM. The resulting microbial floc was incubated for 7 days under light conditions. The final cell concentration of *C. sorokiniana* in the culture broth was determined by cell counting. The experimental conditions are detailed in Table 1. These experiments were performed in triplicate.

**Table 1:** Experimental conditions in examining the effect of coflocculation of *Chlorella sorokiniana* and the symbiont on *C. sorokiniana* growth

Culture type	Mixed microorganism	CaCl_2_ addition	Abbreviation*
Control culture	NA	NA	Control
Mixed culture	CSSB-3	NA	SB
Coflocculated culture	CSSB-3	Added	SBCa

* Abbreviation used in Fig. 5.

NA, not added.

Effects of flocculated symbiont on growth of *C. sorokiniana* were evaluated via anova. Significant results were subjected to Tukey–Kramer's test for multiple comparisons between groups. The calculations were carried out using microsoft excel 2003 with statcel2 as add-in software (OMS Publishing, Tokorozawa, Japan).

### Observation of the *Chlorella*–symbiont complex via SEM

Two hundred and fifty microlitres of *Chlorella*–symbiont complex culture was fixed using 250 μL of 0.2 M sodium cacodylate, 250 μL of 4% (v/v) glutaraldehyde and 250 μL of 0.2 M phosphate buffer (per litre distilled water: 5.28 g NaH_2_PO_4_·H_2_O and 43.46 g Na_2_HPO_4_× 7H_2_O, pH 7.4) and again fixed with 250 μL of 0.2 M sodium cacodylate and 4% osmic acid, and 250 μL of 0.2 M phosphate buffer (pH 7.4). The fixed sample was dehydrated sequentially with 50%, 70%, 80%, 90% and 100% (v/v) ethanol. Finally, 100% ethanol was exchanged with 2-methyl-2-propanol. This sample was freeze-dried (VD-800F, Taitec, Tokyo, Japan), and coated with Pt – Pd (E102, Hitachi, Tokyo, Japan). The sample was observed with a field-emission scanning electron microscope (JSM-6330F, JEOL, Tokyo, Japan) at an accelerating voltage of 5 kV. Micrographs were taken at × 10 000 magnification.

### Isolation and identification of a propionate-degrading bacterium

*Chlorella* culture broth was used for isolation of a propionate-degrading bacterium. The culture broth contained extracellular organic carbon (EOC), which is excreted during *C. sorokiniana* culture (Watanabe *et al.*, 2005). The filtered culture broth was mixed with an equal volume of photoautotrophic medium, and the pH was adjusted to 6.0 with 1 N HCl. One gram of soil sample was incubated in EOC medium containing 1.0 g L^−1^ sodium propionate at 30 °C for 3 days. Thereafter, 1 mL of the culture medium was transferred into fresh EOC medium containing 5.0 g L^−1^ sodium propionate and incubated at 30 °C for 24 h. Bacterial cells were isolated from the resultant culture by spreading and cultivating on an EOC agar plate (2.0% agar) containing 5.0 g L^−1^ propionate for 3 days. A propionate-degrading bacterial strain (PDS1) was obtained from this procedure.

Strain PDS1 was characterized based on 16S rRNA gene sequence analysis. DNA was extracted from samples using the Instagene matrix (Bio-Rad) according to the manufacturer's protocol. Amplification of the 16S rRNA gene was carried out using universal primers 27F and 1492R (primers are listed in Table 2). The PCR products were sequenced directly on a CEQ 2000 DNA sequencer using a CEQ Dye Terminator Cycle Sequencing (DTCS) Quick Start Kit (Beckman Coulter, Fullerton, CA). The 16S rRNA gene sequence primers 27F, 341F, 518R, 530F, 764F, 911R, 1100R, 1390R and 1492R were used. Sequences of all primers in this study are shown in Table 2. The sequences obtained were compared with known sequences using the FASTA service.

**Table 2:** PCR and sequence primers used in identification of a propionate-degrading bacterium

Primer name	Primer sequence (5′–3′)	References
27F	AGAGTTTGATCMTGGCTCAG	Hiraishi (1992)
1492R	GGTTACCTTGTTACGACTT	Hiraishi (1992)
341F	CCTACGGGAGGCAGCAG	Muyzer *et al.* (1993)
518R	ATTACCGCGGCTGCTGG	Muyzer *et al.* (1993)
530F	GTGCCAGCMGCCGCGG	Lane (1991)
764F	CAAACAGGATTAGATACCC	This study
907R	CCGTCAATTCATTTGAGTTT	Lane (1991)
1100R	GGGTTGCGCTCGTTG	Lane (1991)
1390R	ACGGGCGGTGTGTRCAA	Lane (1991)

### Propionate-degrading activity of the isolated bacterial strain

PDS1 was precultured in DLB medium with shaking at 30 °C for 24 h. This bacterial cell suspension was added to fresh DLB medium containing 1 g L^−1^ propionate at an A_580 nm_ of 0.02, and incubated on a rotary shaker (200 r.p.m.) at 30 °C. Propionate concentrations were measured on a liquid chromatograph equipped with Inertsil ODS-3 column (GL Science, Tokyo, Japan) and Intelligent UV/VIS detector (UV-970; Jasco Company, Tokyo, Japan). The oven temperature was maintained at 40 °C, while 20 mM phosphate buffer (containing 2.72 g KH_2_PO_4_ L^−1^; pH adjusted to 3.0 with H_3_PO_4_) was used for elution at a flow rate of 1.0 mL min^−1^.

### Effect of propionate on *Chlorella* growth

The effect of propionate concentration on axenic *C. sorokiniana* culture was examined. Five hundred microlitres of the precultured *C. sorokiniana* (containing 5.0 × 10^7^ cells mL^−1^) was inoculated into 4.5 mL MM4N medium containing the desired concentration of propionate in a Monod tube. It was cultured at 30 °C for 3 days on a monod-type shaker (Monosin II A, Taitec, Saitama, Japan) under a light intensity of 200 μmol m^−2^ s^−1^. The *C. sorokiniana* cell concentration was determined after 3 days of cultivation. This experiment was performed four times and the results were expressed as the mean values.

### Effect of *Chlorella*–PSD1 flocculation on *Chlorella* growth in a medium containing propionate

An appropriate volume of the precultured *C. sorokiniana* was taken to obtain 2.5 × 10^7^ cells, and the volume was adjusted to 450 μL with MM4N medium. Fifty microlitres of the washed PDS1 culture (containing 2.5 × 10^9^ cells) was mixed with the *Chlorella* culture and 500 μL of 500 mM CaCl_2_ solution was added for flocculation. The microbial floc obtained was inoculated to 4 mL MM4N medium containing 125 mg L^−1^ sodium propionate. The *Chlorella* cell concentration was determined on the 3rd day by cell counting. The experimental conditions are detailed in Table 3. These experiments were performed in triplicate. Effects of flocculated PDS1 on *C. sorokiniana* cell growth were evaluated via anova and significant results were subjected to Tukey–Kramer's test for multiple comparisons between groups. Calculations were carried out using microsoft excel 2003 with statcel2 as add-in software (OMS Publishing).

**Table 3:** Experimental condition in examining the effect of coflocculation of *Chlorella sorokiniana* and a propionate-degrading bacterial strain (PDS1) on *C. sorokiniana* growth in a medium containing propionate

Culture type	Mixed microorganism	Propionate addition	CaCl_2_ addition	Abbreviation*
Control culture	NA	NA	NA	Control
Axenic culture	NA	Added	NA	PP
Axenically flocculated culture	NA	Added	Added	PPCa
Coflocculated culture	PDS1	Added	Added	PDSCa

* Abbreviation used in Fig. 8.

NA, not added.

## Results

### Effect of metal concentration on saccharide precipitation from culture broth of *C. sorokiniana*

**Fig. 1:**
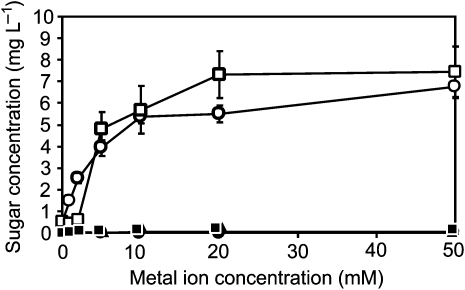
Effect of various metal ions on saccharide precipitation from *Chlorella sorokiniana* culture broth. (▴) NaCl, (•) KCl, (▪) MgCl_2_, (○) CaCl_2_, (□) SrCl_2_. Error bars are ±SE (*n* =9).

The amount of saccharide precipitated from the culture broth increased with increase in the concentration of calcium and strontium chlorides. Maximum amounts of saccharide were achieved when 20 mM of Sr^2+^ or 50 mM of Ca^2+^ was added to the culture broth (Fig. 1). There were no saccharide precipitates when NaCl, KCl or MgCl_2_ was added to the culture.

### Effect of cation on *Chlorella* growth and flocculation

**Fig. 2:**
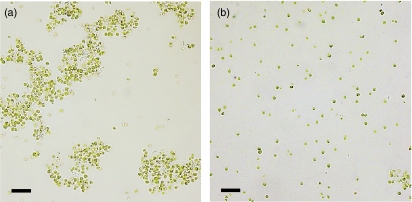
Photomicrograph of cells of *Chlorella sorokiniana* after 1 week of cultivation in a medium containing CaCl_2_. *Chlorella sorokiniana* floc (a) with addition of CaCl_2_ and control culture (b) without CaCl_2_ addition. Scale bar=20 μm.

**Fig. 3:**
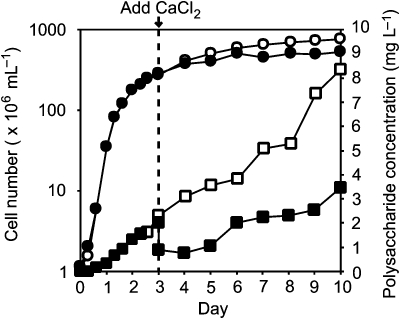
Growth of *Chlorella sorokiniana* and saccharide production in a medium containing CaCl_2_. Growth of *C. sorokiniana* (cell number, circles) and dissolved polysaccharide (squares). Open symbols (○, □) are control culture without addition of CaCl_2_, closed symbols (•, ▪) are with addition of CaCl_2_.

Addition of CaCl_2_ solution resulted in flocculation of *C. sorokiniana*. Each floc comprised tens of *C. sorokiniana* cells within a gelatinous matrix. The *Chlorella*-flocs were observed even 7 days after cation addition (Fig. 2). When CaCl_2_ solution was added to the culture, the amount of polysaccharide in the *C. sorokiniana* culture broth decreased from 2.02 to 0.89 mg L^−1^ (Fig. 3). In the control culture, the quantity of polysaccharide in the culture broth increased linearly, reaching 8.34 mg L^−1^ on day 10. In the Ca^2+^ addition culture, only 3.45 mg L^−1^ of polysaccharide was produced by day 10. The quantity of polysaccharide in the culture with CaCl_2_ was always lower than those in cultures without addition of CaCl_2_. In the flocculated *C. sorokiniana* culture, cell numbers increased from an initial value of 2.85 × 10^8^ to 5.26 × 10^8^ cells mL^−1^ at the end of the culture. However, this value was lower than the value obtained in the control experiment (7.69 × 10^8^ cells mL^−1^).

### Coflocculation of *C. sorokiniana* and the symbiont

**Fig. 4:**
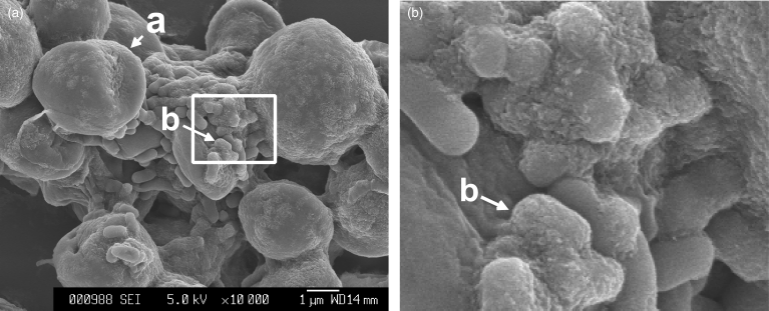
Observation of flocs by SEM. Mixed flocculation of *Chlorella sorokiniana* with CSSB-3 (a). (b) is a magnification of (a).

Coflocculated *C. sorokiniana* and its symbiont were studied as an example of artificial symbiotic microorganisms. *Chlorella sorokiniana* and its symbiont (CSSB-3) were coflocculated within the gel matrix produced by *C. sorokiniana* in a medium containing CaCl_2_. These flocs were observed immediately after addition of CaCl_2_. Electron microscopic observation revealed that *C. sorokiniana* and the symbiont were coflocculated within the algal sheath even 1 week after the addition of CaCl_2_ (Fig. 4).

### Effect of coflocculated symbiont on growth of *Chlorella*

**Fig. 5:**
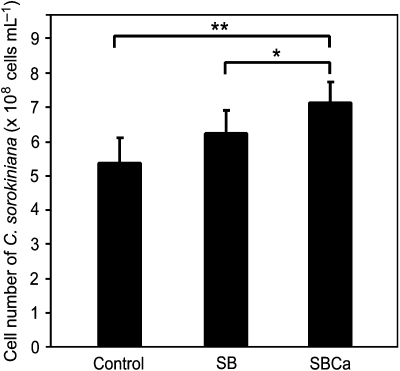
*Chlorella sorokiniana* coflocculated with symbiotic bacteria (CSSB-3) after 1 week of cultivation in a medium containing CaCl_2_. SB, mixed culture; SBCa, coflocculated culture. Error bars are ±SD (*n* = 9). *P* values were determined by the Tukey*–*Kramer test. ^*^Significant difference at *P* <0.05; ^**^significant difference at *P* <0.01.

The number of *C. sorokiniana* cells was counted 7 days after addition of symbionts and CaCl_2_. The *C. sorokiniana* cell number obtained in the coflocculated culture (SBCa, mean 7.14 × 10^8^ cells mL^−1^) was significantly (*P* <0.01) higher than the number obtained in the control culture (Control, mean 5.39 × 10^8^ cells mL^−1^). It was also higher than the number of *C. sorokiniana* obtained in the mixed culture (SB, mean 6.24 × 10^8^ cells mL^−1^, *P* <0.05; Fig. 5).

### Isolation and characterization of a propionate-degrading bacterium

**Fig. 6:**
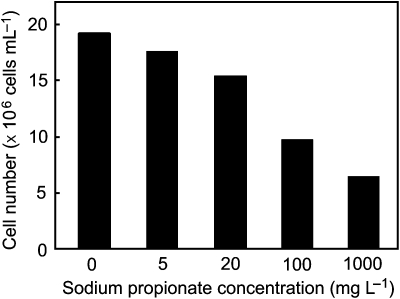
Effect of various concentrations of sodium propionate on growth of *Chlorella sorokiniana*.

**Fig. 7:**
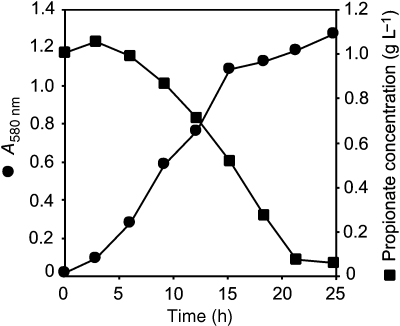
Growth and propionate consumption by PDS1 in DLB medium containing 1 g L^−1^ sodium propionate.

The results of axenic cultivation of *C. sorokiniana* in the medium containing sodium propionate showed that propionate inhibited growth of *C. sorokiniana* (Fig. 6). A complex of *C. sorokiniana* and propionate-degrading bacterium that can grow in the presence of propionate was therefore constructed. Screening for propionate-degrading bacteria was done using the EOC medium containing sodium propionate. The bacterial strain isolated (PDS1) was able to degrade 94% of the propionate in the medium within 24 h (Fig. 7). PDS1 grew in EOC medium with or without propionate, but it did not grow in the photoautotrophic medium containing propionate as the sole carbon source (data not shown). Identification of the bacteria isolated was carried out by 16S rRNA gene sequence analysis. Strain PDS1 (AB302851) showed 99.9% similarity to *Microbacterium oxydans* (AJ717357) belonging to the *Actinobacteria*.

### Effect of coflocculated PDS1 on growth of *Chlorella*

**Fig. 8:**
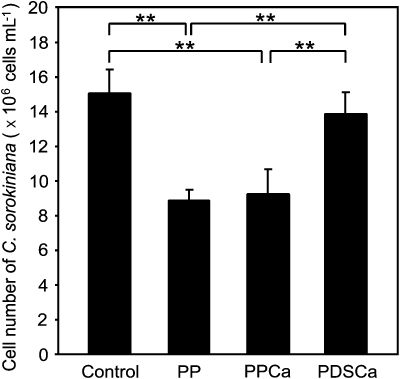
*Chlorella sorokiniana* growth in coflocculated culture with PDS1 after 3 days of cultivation in MM4N medium containing 100 mg L^−1^ sodium propionate. PP, axenic culture; PPCa, axenically flocculated culture; PDSCa, coflocculated culture. Error bars are ±SD (*n* = 9). *P* values were determined by the Tukey*–*Kramer test. ^**^Significant difference at *P* <0.01.

A coflocculated complex of *C. sorokiniana* and PDS1 was constructed by addition of CaCl_2_ to a mixture of PDS1 and *C. sorokiniana*. This floc was cultured in a medium containing propionate, and *C. sorokiniana* cell number was counted on the third day. In axenic culture (PP), average cell *C. sorokiniana* number was 8.9 × 10^6^ cells mL^−1^, which is significantly lower (*P* <0.01) than the 15.1 × 10^6^ cells mL^−1^ obtained in the control culture without addition of propionate (Control; Fig. 8). By contrast, in the coflocculated culture of *C. sorokiniana* and PDS1 (PDSCa), mean cell number (13.9 × 10^6^ cells mL^−1^) was higher than that obtained in axenic culture (PP). There was no significant difference between the cell concentrations obtained in coflocculated culture (PDSCa) and control cultures (Control). At the end of the coflocculated culture, the propionate concentration in the medium decreased below the measurement limits (15 mg L^−1^) of our HPLC system. However, there was no significant decrease in the concentration of propionate in the control culture (data not shown). There was no significant difference in the cell concentrations of *C. sorokiniana* in the axenically flocculated culture (PPCa) and axenic culture (PP).

## Discussion

This study was aimed at developing a method for construction of a close and stable artificial complex of microorganisms. The amount of *C. sorokiniana* sheath was artificially controlled and microorganisms were incorporated into the sheath.

Addition of either CaCl_2_ or SrCl_2_ to the filtered culture broth of *C. sorokiniana* resulted in sedimentation of gelatinous saccharides. The results showed that microorganisms can be incorporated into the gel formed in the *C. sorokiniana* culture broth. We have previously shown that Mg^2+^ is the main metal ion in the *C. sorokiniana* sheath (Watanabe *et al.*, 2006). However, in the present study, addition of MgCl_2_ to *C. sorokiniana* culture broth did not result in saccharide sedimentation. The apparent discrepancy may be due to differences in the affinity between the secreted saccharide and the metal ions. The effects of cations on gelation of saccharide have been extensively studied. For example, it was reported that differences in the type of metal influence the gelation of alginate (Haug & Smidsrod, 1965). The medium used for photoautotrophic cultivation of *C. sorokiniana* contains abundant Mg^2+^. During the cultivation, a part of the saccharide secreted by *C. sorokiniana* was gelled by the abundant Mg^2+^ in the medium, and only those saccharides that were not gelled by magnesium remained in the culture broth. It was these ungelled saccharides that were precipitated by addition of Ca^2+^ or Sr^2+^.

Calcium chloride was used to flocculate *C. sorokiniana* in the present study. Calcium chloride is cheaper than SrCl_2_, and it has only minor detrimental effects on the environment (calcium dichloride is used as a snow melting agent in Japan). Addition of metal solution increased the amount of sheath produced by *C. sorokiniana. Chlorella* cells were immediately flocculated by the gelatinous matter after addition of metal cations while the amount of saccharide dissolved in culture broth decreased. The decrease in saccharide concentration in the broth was equal to the amount of gelled saccharides that adhered to algal cells. On day 10 of culture, a large proportion of *C. sorokiniana* cells were in the gelatinous flocs. The difference in sugar concentrations between the control and the Ca^2+^-supplemented cultures remained constant during the incubation period. These results suggest that saccharide gelation and increase in the *C. sorokiniana* sheath by calcium salt solution continued throughout the period of cultivation. The above results showed that it was feasible to increase *C. sorokiniana* sheath by addition of metal cations. This is the first report of immobilization of microorganisms (flocculation) by addition of metal cations using saccharides produced by the same microorganism.

We constructed a coflocculated symbiotic community of *C. sorokiniana* and its symbiont as an example of an artificial microorganism complex using the immobilization method described here. Strain CSSB-3 is a symbiotic bacterium isolated from *C. sorokiniana* culture. This bacterium promoted the growth of *C. sorokiniana* (Watanabe *et al.*, 2005). Using this bacterium as a model microorganism, we constructed a close symbiotic community within the extracellular polysaccharide augmented artificially by addition of metal solution. Electron microscopic observation of the constructed floc showed that the symbionts were attached to the surface of *C. sorokiniana* cells by gelatinous matter. The enhanced growth of *C. sorokiniana* in the *Chlorella* and CSSB-3 coflocculated culture is an indication that attachment through algal sheath enhances interaction of *C. sorokiniana* and the symbiont. It was reported that coimmobilization of *Chlorella* and plant-growth-promoting bacteria (PGPR) within the same alginate beads enhanced algal growth and pigment production (Gonzalez & Bashan, 2000). These authors reported that keeping the microorganisms close together was effective in promoting interactions between them. The coflocculation method described herein is a simple means of facilitating interactions between *Chlorella* and its associated microorganisms.

We employed the same method for coflocculation of *C. sorokiniana* and microorganisms isolated from the environment, and showed that the method is applicable to various microorganisms. An application of this method was demonstrated by constructing a complex of *C. sorokiniana* and propionate-degrading bacteria (PDS1). The complex can degrade propionate. Propionate, depending on its concentration, inhibited *C. sorokiniana* growth (Fig. 6). In the *Chlorella–*PDS1 complex, *C. sorokiniana* was able to grow in the presence of a high concentration of propionate. This complex was able to degrade propionate in the medium. When strain PDS1 was cultivated on EOC medium, it grew well with or without propionate (data not shown). In terms of nutrient utilization under photoautotrophic conditions, PDS1 depends on EOC released by *C. sorokiniana*. In microalgal*–*bacterial symbiotic associations, the microalgae generally provide O_2_ and photosynthetic products to bacteria, and bacteria provide CO_2_ by degrading photosynthate. Moreover, the bacteria also act as algal growth-promoters (Munoz & Guieysse, 2006). It was reported that indole-3-acetic acid, produced by *Azospirillum brasilense*, worked as a growth factor when *Chlorella vulgaris* was coimmobilized and cocultured in alginate beads with *A. brasilense* (Gonzalez & Bashan, 2000). In the coflocculated complex of *Chlorella* and PDS1, the following mutualisms exist under light conditions: *C. sorokiniana* provides PDS1 with photosynthetic products, while PDS1 degrades propionate which inhibits algal growth, and also provides CO_2_ to *Chlorella* by degrading EOC and propionate. However, we have not clarified the growth factor in this study.

*Chlorella sorokiniana* has the ability to remove elements contained in high-concentration wastewater except the propionate (Ogbonna *et al.*, 2000). This *Chlorella–*PDS1 complex can be used for treatment of wastewater containing propionate without addition of extraneous nutrients. It may be feasible to construct symbiotic communities of *C. sorokiniana* and suitable microorganisms for various applications. Production of extracellular polysaccharides is a general phenomenon in photosynthetic microorganisms (Lewin, 1956; O'Colla, 1963). In addition, it is known that the gellan gums produced by *Pseudomonas* sp. form gels in the presence of cations (Kang *et al.*, 1982). The method described herein can be applied to a wide range of algae, and can also be applied to heterotrophic microorganisms.

Several methods for immobilization such as adsorption, entrapment and coupling have been developed (Kolot, 1981). Our method has some analogy with the entrapment method, but it is unique in constructing the carrier using the immobilized microorganism itself. In this method, only addition of metal solution is required for cell immobilization. It is therefore simple compared with the other methods. In addition, the number and size of the aggregation increase with the culture process as long as the metal cation exists in the culture broth and the microorganism produces the soluble polysaccharide. By contrast, the properties of the carrier depend on the polysaccharide produced by microorganism. Therefore, cell leakage and a physical vulnerability might become problems dependent on the type of microorganism. Also, it is difficult to control the size of the flocculated complex. To meet the requirements for an ideal immobilization matrix (Mallick, 2002) further study is necessary. In conclusion, a simple procedure for construction of close and stable artificial communities of microorganisms is proposed in this study.

## Statement

Re-use of this article is permitted in accordance with the Creative Commons Deed, Attribution 2.5, which does not permit commercial exploitation.
